# Effect of a High-Protein, High-Fiber Beverage Preload on Subjective Appetite Ratings and Subsequent Ad Libitum Energy Intake in Overweight Men and Women: A Randomized, Double-Blind Placebo-Controlled, Crossover Study

**DOI:** 10.1093/cdn/nzy022

**Published:** 2018-06-23

**Authors:** Mastaneh Sharafi, Nima Alamdari, Michael Wilson, Heather J Leidy, Erin L Glynn

**Affiliations:** 1 Research and Development, Beachbody, LLC, Santa Monica, CA; 2 Scientific Affairs, Beachbody, LLC, Santa Monica, CA; 3 Department of Nutrition Science, Purdue University, West Lafayette, IN

**Keywords:** dietary protein, dietary fiber, satiety, appetite, energy intake, food intake, dietary restraint, preload, dietary supplement

## Abstract

**Background:**

Dietary protein and fiber have been shown to independently improve subjective measures of appetite control.

**Objective:**

The aim of this study was to determine the acute effects of a high-protein, high-fiber (HP/HFb) beverage taken as a preload compared with an isocaloric lower-protein, lower-fiber (LP/LFb) placebo beverage on subjective appetite ratings and subsequent energy intake at an ad libitum meal in healthy adults.

**Methods:**

A total of 50 overweight/obese men and women [*n* = 25 men, 25 women; age 30 ± 2 y; body mass index (BMI) 29.6 ± 0.3 kg/m^2^] received a 160 kcal HP/HFb beverage containing 17 g protein and 6 g fiber on one occasion and an isocaloric LP/LFb placebo beverage containing 1 g protein and 3 g fiber on another occasion in a randomized, double-blind, crossover design. Thirty min after consumption of the beverage preload, an ad libitum pizza meal was provided to be consumed over a 30-min period. Visual analog scales (VAS) were used to assess subjective appetite ratings throughout the testing period. The Revised Restraint Scale (RRS) was used to classify participants as restrained or unrestrained eaters.

**Results:**

HP/HFb led to greater reductions in postprandial desire to eat and hunger compared with LP/LFb (both, *P* < 0.05) but did not significantly affect postprandial fullness or prospective food consumption. Subsequent meal energy intake tended to be lower after HP/HFb compared with LP/LFb (*P* = 0.09). A subanalysis showed lower energy intake after HP/HFb in older participants (≥25 y) compared with LP/LFb, which was not observed in the younger participants (<25 y).

**Conclusions:**

Compared with LP/LFb, a HP/HFb beverage preload reduced hunger, desire to eat, and tended to reduce subsequent food intake. Dietary restraint and age appear to influence subsequent energy intake and should be taken into account when designing nutrition interventions for weight reduction and/or maintenance. This trial was registered at clinicaltrials.gov as NCT02979717.

## Introduction

Overweight and obesity continue to be major public health concerns, with the latest statistics showing over 70% of US adults age 20 and over are overweight, and 37.9% are obese (NHANES 2013–2014) (1). Worldwide, more than 1.9 billion adults are overweight and 650 million are obese (WHO 2017) (2). In an effort to combat the obesity epidemic, numerous dietary approaches have been proposed for weight loss and subsequent weight maintenance. One such approach has been the manipulation of macronutrient ratios, specifically increasing the proportion of protein in relation to carbohydrates and/or fat.

There is strong support for dietary protein improving acute appetite control, and this effect is greater than that of carbohydrates and/or fats (3–4). In addition, fiber has been shown to have similar acute effects on measures of appetite, but these do not necessarily translate into reductions in energy intake (5). Interestingly, recent research suggests that combining fiber with lower amounts of protein in solid food forms elicits a similar effect on appetite as high protein alone (6–7). Improved appetite control may facilitate weight loss and weight management by helping to improve compliance of individuals on energy-restricted diets (4). However, many other contributing factors including age (8), gender (9, 10), weight status, and dietary restraint (11) can influence outcomes related to appetite control and energy intake.

Consumers have an increased desire to consume more “on-the-go” foods containing protein and/or fiber (12); however, limited evidence exists assessing the potential synergistic effects of protein and fiber for appetite control. The objective of this study was to determine the acute effects of a high-protein, high-fiber (HP/HFb) beverage taken as a preload on subjective appetite ratings and subsequent energy intake at an ad libitum meal compared with an isocaloric lower-protein, lower-fiber (LP/LFb) placebo.

## Methods

This trial was registered at clinicaltrials.gov (NCT02979717), and additional information including full inclusion/exclusion criteria may be found there. The study was conducted by an independent laboratory (GI Labs, Toronto, Ontario, Canada) and approved by the Western Institutional Research Board, which meets all the requirements of the US FDA, the Department of Health and Human Services (DHHS), the Canadian Health Protection Branch (HPB), the Canadian Institute of Health Research (CIHR), and the European Community Guidelines. All participants gave their voluntary consent by signing the consent form after the experimental procedure had been explained to them verbally and in writing. After gaining consent, information was collected at screening on demographic characteristics, medical history, and eating behavior. Participants were compensated $60 for completing all study procedures.

**FIGURE 1:**
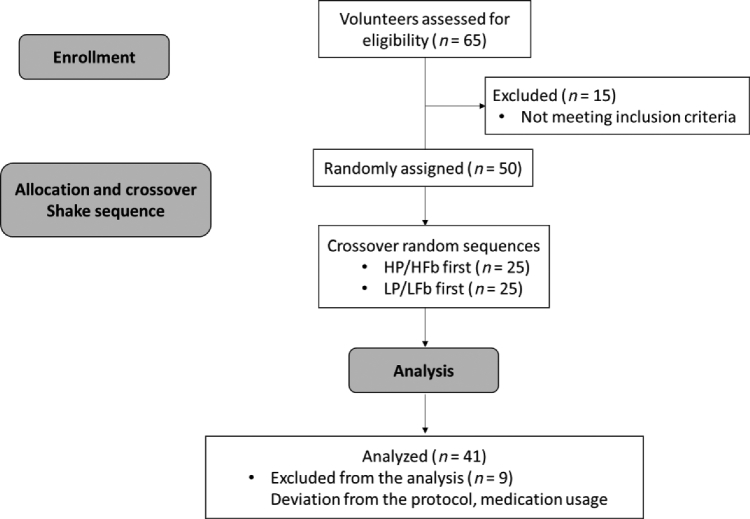
Consolidated Standards of Reporting Trials (CONSORT) flow diagram of the recruitment, enrollment, and random assignment processes. HP/HFb: high-protein/high-fiber; LP/LFb: lower-protein/lower-fiber.

### Participants

Fifty adults (men, *n* = 25 and women, *n* = 25) were recruited between November 2016 and February 2017 in Toronto, Ontario, Canada. Inclusion criteria included: age 18–50 y, overweight/obese individuals (BMI range: 27–33 kg/m^2^), non-smokers, willingness to abstain from strenuous exercise, alcoholic drink consumption, and caffeine-containing foods/beverages 24 h before the study days and during study days, and no previous history/presence of diabetes, hypertension, cardiovascular, pulmonary, biliary, and gastrointestinal disorders or liver disease. Exclusion criteria included: history of eating disorders, pregnancy or breastfeeding, use of medications known to influence carbohydrate metabolism, gastrointestinal function, or appetite, major trauma or surgical event within 3 mo of screening, known intolerance, sensitivity, or allergy to any ingredients in the study product, and change in body weight >3.5 kg within 4 wk of the screening visit. Women were tested during the luteal phase of their menstrual cycle.

A power calculation was performed and determined 40 participants were required to detect a 700 KJ (167 kcal) difference in energy intake with 80% statistical power and *α* = 0.05 (13–14). Of the 50 participants enrolled, 41 accurately completed all study procedures. Four subjects (all women) deviated from the allotted time per protocol to consume preload beverages, which could have a significant effect on responses given the short timeline of the study. Five (2 women, 3 men) reported taking anxiety/depression medications (**Figure 1**). Subject characteristics are presented in **Table 1**.

**TABLE 1:** Subject characteristics (*n* = 41)^1^

	All	Men (*n* = 22)	Women (*n* = 19)
Age, y	30 ± 2	31 ± 2	29 ± 2
Ethnicity, % (Caucasian/other^2^)	51/49	50/50	53/47
Body weight, kg	87 ± 2	92 ± 2	82 ± 3
BMI, kg/m^2^	29.6 ± 0.3	29.2 ± 0.4	29.9 ± 0.4
Waist, cm	96 ± 1	99 ± 2	92 ± 2
Dietary restraint score	11.8 ± 1	10.2 ± 1	13.5 ± 1^*^

1 Data are presented as mean ± SEMs.

2 ^2^Other includes Asian, Black, Arab/Western Asian, and Hispanic/Latino.

* *P* < 0.05.

### Experimental design

This was a double-blind, placebo-controlled, crossover study design consisting of 2 beverages taken 30 min before a meal: HP/HFb and LP/LFb placebo. Participants were randomly assigned to consume either HP/HFb or LP/LFb at Visit 1, followed by the opposite beverage at Visit 2 (Figure 1). Separate randomization schedules were used for men and women to ensure balance within sequences. Randomization schedules were generated using SAS Version 9.4 for PC (SAS Institute Inc., Cary, NC), procedure PLAN. The program was seeded with a random number start seed. Subjects were randomized to 1 of 2 test sequences: Test Shake first period followed by Control Shake second period (TC) or Control Shake first period followed by Test Shake second period (CT). The schedules were blocked by 2 so that after every 2 subjects randomized the sequences were balanced. Each randomization schedule was independently seeded. There was a minimum 7-d washout period between visits (maximum of 22 d).

Subjects were asked to maintain their habitual diet, physical activity pattern, and body weight throughout the study. Participants were asked to eat approximately the same type and size of dinner the evening before each test day. The morning of a test day, subjects were asked what time they finished their last meal and if they “engaged in unusual levels of food intake the night before”. Prior to the start of the study visit, there was a diet record recall for “previous evening dinner” filled out by study staff.

**FIGURE 2:**
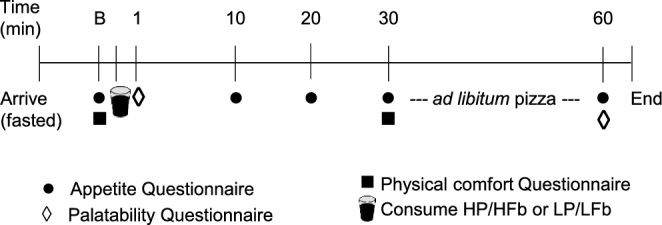
Testing day procedures. B, baseline; HP/HFb: high-protein, high-fiber; LP/LFb: lower-protein, lower-fiber.

Testing day procedures are shown in **Figure 2**. Subjects arrived to the testing facility after a 10–12-h overnight fast. Prior to consumption of the assigned beverage preload (baseline: B), subjects completed appetite and physical comfort questionnaires. Subjects were then instructed to consume the beverage within 5 min. Immediately after the consumption of the beverage preload, a palatability questionnaire was completed, followed by the appetite questionnaire at 10, 20, and 30 min post consumption. Also at t 30 min, subjects completed a physical comfort questionnaire. Subjects were then provided an ad libitum pizza meal and asked to consume pizza over the next 30 min until they were comfortably full. At 60 min, final appetite and palatability questionnaires were completed and the participants were permitted to leave the facility. We chose to serve the pizza meal 30 min after beverage ingestion based on a pilot study in which we found peak appetite effects of the HP/HFb beverage at 30 min in a 3-h study period. Differences in most appetite responses between HP/HFb and LP/LFb were also greatest at 30 min.

### Blinding

Products were provided in blank sachets, except for a printed study code. A letter was provided to the testing facility in a sealed, opaque envelope indicating which code was placebo and which was active product. This envelope was readily available for the investigator to open in the event that it became necessary to know which product a participant was taking for the sake of their health care. There were no cases where the investigators needed to be unblinded for this study.

### Test beverages and ad libitum lunch

The nutritional compositions of the test beverages are shown in **Table 2**. The HP/HFb beverage was a commercially available dietary supplement (Shakeology Chocolate flavor, Beachbody LLC, Santa Monica, CA). The dietary supplement is composed of a protein blend (whey, pea), and fruit, vegetable, and plant powders, as well as vitamins, minerals, pre-, and probiotics. Fiber sources include cocoa, pea fiber, xanthan gum, inulin, flaxseed, chicory root, and chia seed powder. The maltodextrin-based, flavor-matched isocaloric LP/LFb placebo beverage was manufactured for this trial. As 2 main ingredients believed to contribute to satiety effects of the HP/HFb beverage are protein and fiber, the placebo was designed to omit protein and as much fiber as possible. However, the cocoa and xanthan gum included in the placebo contributed some fiber.

**TABLE 2:** Nutritional composition of beverage preloads^1^

Preload	HP/HFb	LP/LFb
Calories, kcal	160	160
Protein, g	17	1
Fat, g	2	0.5
Carbohydrate, g	17	37
Sugar, g	7	13
Fiber, g	6	3
Beverage volume, oz	10	10
Palatability ± SEM, mm	48 ± 3	56 ± 3

1 HP/HFb: high-protein/high-fiber; LP/LFb: lower-protein, lower-fiber.

The ad libitum pizza meal consisted of individual pizzas (Giuseppe Pizzeria Mini Pizzas, Dr. Oetker, Mississauga, Ontario, Canada). Participants were allowed to choose either cheese or pepperoni pizza, but received the same type at both study visits. Pizza was served in a private room, where 3 plates of rectangular pizza slices were served at 10-min intervals. Each plate offered contained 4 pizzas each for a total of 12 personal pizzas offered over the 30-min period. The total energy content offered to each subject was 2280 kcal. Nutrition information for the pizzas is as follows: Cheese: 1 pizza (82 g) = 190 kcal, 6 g fat, 24 g carbohydrate, 9 g protein; Pepperoni: 1 pizza (87 g) = 190 kcal, 6 g fat, 24 g carbohydrate, 9 g protein. Subjects were also given a 1.5 L bottle of mineral water and were instructed to eat and drink as much as they desired until they were “comfortably full”.

### Appetite, palatability, and physical comfort ratings

Subjective measurements of appetite (i.e., hunger, fullness, desire to eat, prospective food consumption), palatability (i.e., pleasantness of food, taste, smell, appearance, mouthfeel, and aftertaste), and physical comfort (i.e., bloating, nausea) were assessed using visual analog scales (VAS) (15). Each of the questions included a 100-mm VAS anchored at each end with opposing statements. Participants marked a vertical line on the line at a point that they felt reflected their feelings at the moment the test was taken. Scores were assessed by measuring the distance between the intersection of the vertical line with the line and the left end of the line. Subjects were not permitted to refer to their previous ratings when completing the VAS. A composite appetite score (CAS) was calculated as: CAS = (desire to eat + hunger + Prospective Consumption + (100 – Fullness)) / 4 (16). An overall palatability rating was formed by taking an average of the palatability indicators.

### Dietary restraint

The Revised Restraint Scale (RRS) was used to assess total dietary restraint at screening (17). RRS is a 10-item measure used for identifying restrained eaters. Items of RRS are rated on a 4–5-point scale, with a maximum total score of 35 and including 2 subscales: (a) concern for dieting, with 6 items to measure the attitudes towards dieting; and (b) weight fluctuation, with 4 items to measure history of weight fluctuation. Higher scores on the RRS indicate higher levels of dietary restraint. Typically, a median split has been used to create groups of restrained and unrestrained eaters of approximately equal size in similar research, with the most recent cutoffs in the 12–14 range, with the average score for normal-weight women being 13 (18). We also used the median cutoff in this study, which was similar to other studies, generating groups of dietary restraint score >13 (restrained) and ≤13 (unrestrained). The Cronbach's alpha coefficient was 0.73.

### Post meal intake

Pizza intake was measured by weighing each plate of pizza immediately before it was served and then weighing the remaining pizza on each plate after it was removed from the room. Water was served in bottles, which were weighed before and after consumption. Total energy consumed was calculated by converting the weight consumed to the calories provided by the manufacturer.

### Data and statistical analyses

A power analysis was conducted before the start of the study to identify the appropriate sample size. Studies on food intake have measured the range of intake at 30 min post load to be 150–358 kcal (13–14). A sample size of 40 subjects (20 per sequence) was sufficient to detect a 167 kcal difference in intake between products with 80% statistical power (*α* = 0.05).

Data were analyzed using SPSS 22.0 software (SPSS, Inc., Chicago, IL). For appetite VAS response, net incremental area under the curve (∆AUC) was calculated over the 30 min from B to before the introduction of the meal using the trapezoidal method (19). We examined the 30 min AUC (instead of the 60 min AUC) to look at the isolated effects of beverage, excluding the effect of subsequent ad libitum pizza consumption. Changes in appetite across time [analysis of change score; 4 time points from B to t 60 (post pizza)] were analyzed by repeated-measure ANCOVA with product and time as within-subject factors and age and sex as covariates. The Greenhouse-Geisser correction was used to adjust for violations of sphericity. Paired *t* tests were performed to investigate the differences in post pizza VAS scores (∆ B to t 60) from HP/HFb to LP/LFb treatment. Ad libitum energy intake at the subsequent meal was analyzed using a mixed-effects model to control for the possible influence of sequence and period on energy intake, where fixed factors were sequence, period, and product (HP/HFb or LP/LFb), the random factor was subject within sequence, and the adjusted model covariates were gender, dietary restraint, and B CAS. Pearson correlation analysis was performed to determine the strength of associations between covariates and calorie intake as well as between age and dietary restraint.

Based on findings from a previous pilot study, and supported by literature (20–22), exploratory subanalyses of VAS and energy intake were conducted to identify differences in the magnitude of beverage effect among participants with different dietary restraint [based on the median cutoff: dietary restraint score >13 (restrained) and ≤13 (unrestrained)] and by age (based on the median cutoff: <25 y and ≥25 y). The separate effects of age and dietary restraint on VAS scores (all time points) were tested by repeated-measure ANOVA. Afterwards, paired *t* tests were performed to investigate the differences in post pizza VAS scores (∆ B to t 60) between HP/HFb and LP/LFb treatments, and mixed-effects analysis was performed to test the effect of product on energy intake in separate groups of dietary restraint and age.

A significance level of *P* < 0.05 was used for all statistical tests. Values presented in graphs are mean ± SEM. No data were transformed.

## Results

### Appetite, palatability, and physical comfort ratings

**FIGURE 3:**
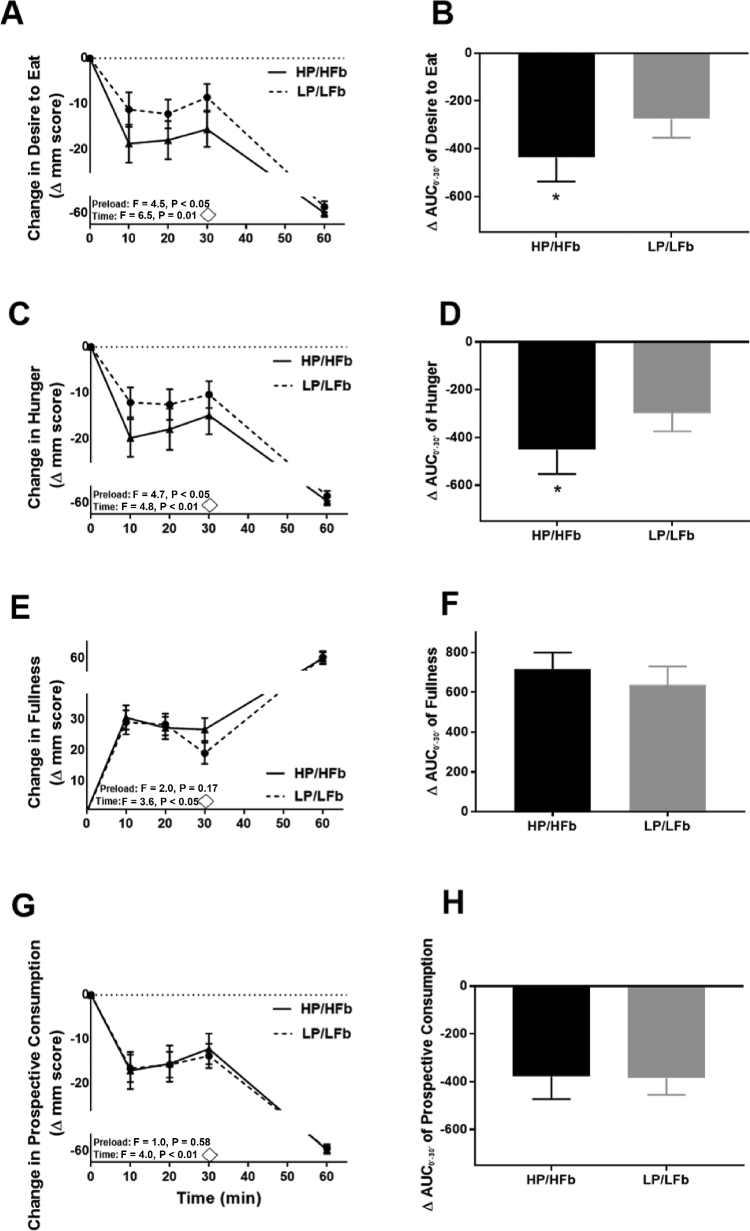
Appetite response time course and 30 min area under the curve (AUC) change in desire to eat (A, B), hunger (C, D), fullness (E, F), and prospective consumption (G, H) following high protein/high fiber (HP/HFb) and isocaloric lower protein/lower fiber placebo (LP/LFb) preloads. ◊ Pizza meal served. Time 60 represents post-meal appetite rating. Values are mean ± SEM. * *P* < 0.05.

Appetite questionnaire responses throughout the testing period are presented in **Figure 3**. There were significant main effects of beverage preload type and time (B to 60 min) on change in desire to eat (Figure 3A). Specifically, although the overall main effect of beverage showed reduced desire to eat throughout the postprandial period, HP/HFb led to greater reductions compared with LP/LFb (*P* < 0.05). When examining 30 min ∆AUC to isolate the beverage effect, desire to eat was also lower after consumption of HP/HFb compared with LP/LFb (*P* < 0.05) (Figure 3B). There were significant main effects of beverage type and time on change in hunger (Figure 3C). Although the main effect of beverage showed reduced hunger throughout the postprandial period (*P* < 0.01), HP/HFb led to greater reductions compared with LP/LFb (*P* < 0.05). The 30 min ∆AUC for hunger was also lower after consumption of HP/HFb compared with LP/LFb (*P* < 0.05) (Figure 3D).

There was a significant main effect of time for fullness and prospective consumption, but no differences between beverages (Figure 3E, G). There were no differences in 30 min ∆AUC for fullness or prospective consumption (Figure 3F, H). When the appetite ratings were combined into a composite score (i.e., CAS), there was a significant main effect of beverage (*P* < 0.05) and time (*P* < 0.0001) with HP/HFb leading to greater reductions in CAS over time compared with LP/LFb. However, the 30 min ∆AUC for CAS was not different between beverages (data not shown). No significant main effect of gender, beverage type-by-gender interactions, or beverage type-by-time interactions were detected for any outcomes.

Paired *t* tests of change score from B to 60 min for desire to eat, hunger, and CAS did not show significant differences between HP/HFb and LP/LFb. There were no significant differences in overall palatability (*P* = 0.4, Table 1) or its indicators pleasantness of food (*P* = 0.20), taste (*P* = 0.41), smell (*P* = 0.59), appearance (*P* = 0.66), mouthfeel (*P* = 0.87), and aftertaste (*P* = 0.29) after consumption of study beverages. There were no differences between HP/HFb and LP/LFb on any of the physical discomfort indicators from min 0 to min 30.

### Analysis of intake

Looking at the ad libitum energy intake in the unadjusted model showed no significant differences between HP/HFb and LP/LFb (860 ± 64.2 compared with 945 ± 64.2). When controlling for factors that were significantly correlated with energy intake (i.e., gender, *r* = 0.52, *P* < 0.0001; B CAS, *r* = 0.24, *P* < 0.05; and dietary restraint, *r* = –0.31, *P* < 0.01), ad libitum energy intake at the subsequent meal tended to be less after the HP/HFb compared with the LP/LFb beverage (857 ±53.1 kcal compared with 946 ± 53.1 kcal, respectively; *F* = 3.0, *P* = 0.09).

### Subanalyses including dietary restraint effects

Exploratory analysis of pilot data after commencement of this trial indicated a potential role of dietary restraint in appetite response to the HP/HFb and LP/LFb beverages. Therefore, participants were retrospectively grouped according to high and low dietary restraint (>13 and ≤13 RRS score, respectively). Chi-square analysis showed no significant difference for the distribution of men and women across the restraint groups (*P* = 0.17), but total dietary restraint score was significantly greater for women (*P* < 0.01; Table 1). No main effects of beverage type or time were detected for the VAS appetite responses throughout the testing period when grouped according to dietary restraint (data not shown). We understand that conservatively, post hoc analyses are conducted on significant main effects. However, since this analysis was exploratory, we believe examining the post hoc results contributes to our overall understanding of the current results, and could potentially provide useful information for the design of future studies and interpretation of other research results. Those with restrained eating displayed a smaller reduction in postprandial desire to eat (*P* = 0.01) and tended to have a smaller reduction in postprandial hunger (*P* = 0.07) and postprandial CAS (*P* = 0.08) after the meal after the LP/LFb beverage compared with the HP/HFb.

No significant main effects of dietary restraint or the interaction between beverage type and dietary restraint were detected for energy intake. When examined individually, HP/HFb significantly reduced energy intake compared with LP/LFb in the unrestrained group (*F* = 5.45, *P* < 0.05), but was not different in the restrained group (*F* = 0.53, *P* = 0.48).

In our sample, dietary restraint score was inversely correlated with age (*r* = –0.59, *P* < 0.0001) such that an increase in age was associated with a reduction in dietary restraint. Based on these findings, an exploratory analysis by age was performed on study outcomes, separating groups by median age: <25 y (*n* = 19, mean age = 22, median = 22) and ≥25 y (*n* = 22, mean age = 32.5, median = 36). In a chi-square analysis, 17 of 22 (77%) individuals ≥25 y were classified as unrestrained, while 14 of 19 (73%) individuals <25 y were classified as restrained (*P* < 0.01). There was a main effect of age group on total restraint score (*F* = 7.6, *P* < 0.01). Looking at the 2 subcomponents of dietary restraint, only concern for dieting was significantly predicted by age group (*F* = 12.3, *P* = 0.001), with the <25 y group reporting higher concern for dieting than the older group. The weight fluctuation subcomponent was not associated with age group (*F* = 1.1, *P* = 0.3).

**FIGURE 4:**
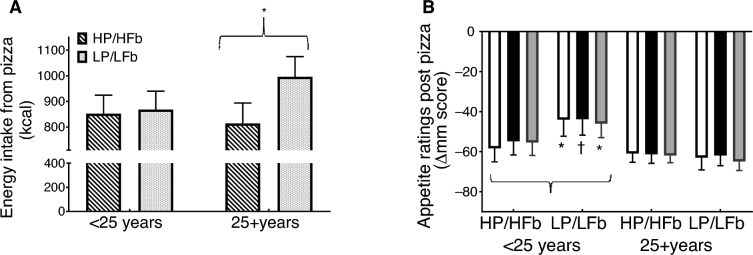
Change in energy intake at ad libitum pizza meal by age (A); and change in desire to eat (white bars), Hunger (black bars), and composite appetite score (gray bars) from baseline to 60 min (post pizza) by age (B) after high-protein/high-fiber (HP/HFb) and lower-protein/lower-fiber (LP/LFb) preloads. Values are mean ± SEM; *n* = 41. † *P* < 0.1, * *P* < 0.05, ** *P* < 0.01.

Due to the strong correlation of restraint with age, a subanalysis by age group was conducted, similar to the dietary restraint analysis. Although no main effects of beverage type and age were detected, a beverage type-by-age interaction was detected for energy intake (*F* = 4.2, *P* < 0.05), desire to eat (*F* = 7.8, *P* < 0.01), and CAS (*F* = 6.4, *P* < 0.05) after the ad libitum meal. Energy intake at the subsequent meal was significantly lower after the HP/HFb beverage compared with the LP/LFb among individuals ≥25 y (*F* = 7.2, *P* = 0.015), but not for those <25 y (**Figure 4A**). Specifically, those <25 y displayed a smaller reduction in desire to eat and CAS after the meal (*P* < 0.01 and *P* < 0.05, respectively), and tended to have less reduction in hunger (*P* = 0.075) post meal after LP/LFb compared with HP/HFb (Figure 4B), whereas those ≥25 y did not (all *P* values >0.3).

## Discussion

This study investigated the effects of a HP/HFb beverage preload containing 17 g protein and 6 g fiber, taken 30 min before a meal, on subjective appetite ratings and subsequent energy intake compared with an isocaloric LP/LFb beverage containing 1 g protein and 3 g fiber. The consumption of the HP/HFb beverage acutely reduced perceived hunger and desire to eat compared with the LP/LFb. In addition, consuming the HP/HFb beverage led to greater reductions in hunger, desire to eat, and CAS over the 60-min study period including a 30 min ad libitum pizza meal compared with the isocaloric LP/LFb beverage. Although there was a trend for reduction in energy intake at an ad libitum pizza meal after the HP/HFb beverage compared with the LP/LFb in the overall study group, exploratory analysis showed dietary restraint and age may influence energy intake. Taken together, these data suggest that compared with a lower-protein, lower-fiber beverage, a beverage high in protein and fiber may be a good strategy to reduce appetite and potentially reduce energy intake at a subsequent meal in specific populations (such as individuals >25 y of age).

Recent evidence has suggested that ∼30 g of protein is sufficient to improve postprandial satiety (4). Yet, the current study illustrated improvements in select markers of appetite (desire to eat and hunger) after the HP/HFb beverage that contained nearly half as much protein. Douglas et al. (6) compared beef and soy proteins, showing that 14 g protein with 5 g fiber elicited appetite responses similar to 24 g protein, and suggesting a possible synergistic effect of fiber and protein on appetite. Therefore, although the amount of protein in the HP/HFb beverage used in the current study was lower than typically seen in protein preload studies, the combination with high fiber likely contributed to the observed improvement in appetite responses.

Despite we observed improvements in the appetite markers of desire to eat and hunger, the current literature to date has illustrated reductions in postprandial hunger, desire to eat, and prospective food consumption along with increases in postprandial fullness after the consumption of higher compared with lower protein preloads. However, increased postprandial fullness has been reported to be the most consistent response compared with the other perceived sensations (4, 23), and similar findings also exist with dietary fiber (24–25). One potential explanation for the difference in findings is the beverage form of preload in this study (26). Owing to the reduced appetite-related hormonal responses after the consumption of beverages compared with solid foods (27), it is possible that the sustained satiating (i.e., fullness) effects of protein and/or fiber were blunted due to the preload food form. The reduction in hunger is more immediate and thus, this response may not have been impacted.

Despite the improvements in appetite, the HP/HFb beverage only tended to reduce energy intake compared with the LP/LFb version. A review of acute feeding trials has also shown that protein meals or preloads have modest satiety effects, but translation to significant reduction in energy intake at the next eating occasion is not always present (4). Potential contributors in the current study could include the preload form of a beverage compared with a solid, and/or the relatively low amount of calories in the preloads compared with other studies (160 kcal compared with typically ≥300 kcal). Similar to most of the acute feeding trials, we did not assess changes in daily energy intake, and thus, it is possible that energy compensation occurred later throughout the day. Another explanation for lack of statistically significant energy intake reduction after the beverages is inclusion of individuals that might have less precise or blunted energy compensation mechanisms (20). Dietary restraint is defined as a tendency to consciously restrict or control food intake. Previous research on fat preloads (with and without carbohydrates) has shown that individuals with restrained eating usually consume the same amount of calories regardless of variations in the preload, and thus, may not show compensatory behaviors compared with unrestrained eaters (20–21). Consistent with this, restrained eaters in this study consumed a similar amount of calories at the meal after either beverage, while the exploratory analysis showed unrestrained eaters reduced energy intake after HP/HFb compared with LP/LFb.

In the current study, dietary restraint was negatively associated with age. This relationship has not been widely investigated, and the results are mixed depending on the scale used to measure dietary restraint (28–29). Our findings are consistent with previous research showing dietary restraint measured by RRS decreased with age (22). When we divided our sample at median age for subanalysis, the majority of younger individuals (age <25 y) were restrained eaters and reported greater concerns for dieting than the older individuals (≥25 y). Subsequently, those ≥25 y consumed significantly more pizza after LP/LFb compared with HP/HFb, while those <25 y controlled their consumption to the same amount regardless of the beverage type. It is possible that higher concerns for dieting and higher dietary restraint in the younger group contributed to the reduced intake at the next eating occasion. Further analysis showed the <25 y group still had a greater desire to eat and greater CAS after the ad libitum meal during the LP/LFb compared with the HP/HFb testing day. Since participants were asked to eat until they were comfortably full, post meal appetite indicators should have been similar across treatments. These findings, while speculative, suggest that meal energy intake in the <25 y group was not in response to internal appetite cues, but possibly a product of dietary behaviors and beliefs.

In this study, the meal was provided 30 min after the preload, as we previously identified peak appetite responses for the shakes at this time. However, the time between a preload and meal in similar study types is often longer. It is possible a longer time period between preload and pizza meal and/or monitoring subsequent energy intake for a greater period of time (i.e., 12–24 h) would have allowed us to further differentiate effects of the 2 test beverages. As previously mentioned, the calorie content of the preloads was also relatively low. Moreover, despite the high fiber content of the HP/HFb preload, the placebo (LP/LFb) also contained a substantial amount of fiber (3 g) due to the cocoa and xanthan gum content. We therefore did not expect to be able to detect differences in appetite hormones, so physiological responses were not measured.

In summary, in overweight adults, consuming a beverage with high protein (17 g) and high fiber (6 g) as a preload improved appetite measures of desire to eat, hunger, and CAS over the study period and tended to reduce energy intake at a subsequent meal compared with a lower-protein (1 g), lower-fiber (3 g) placebo. We found energy intake was influenced by age in this study, likely driven by differences in dietary restraint between those <25 and ≥25 y of age. Although younger individuals consumed a similar amount of calories at the meal after both beverages, post meal subjective appetite ratings suggest they still had a desire to eat and thus utilized restraint to control the amount of calories they consumed. Dietary restraint, and potentially age, influence energy intake after a modest beverage preload and should be taken into account when designing preload studies or dietary interventions for weight reduction and/or maintenance.

## Notes

Supported by Beachbody, LLC.

Author disclosures: MS, NA, MW, and ELG are employees of Beachbody, LLC. HJL has served as an expert consultant for Beachbody, LLC on other solicited assignments. HJL was not compensated for contributions to this work.

Abbreviations used:

B: baseline
CAS: composite appetite score
HP/HFb: high-protein, high-fiber
LP/LFb: lower-protein, lower-fiber
RRS: Revised Restraint Scale
VAS: visual analog scale
